# Innovating Crisis Care: A Multi-Agency Model for High Intensity Users

**DOI:** 10.1192/bjo.2026.11542

**Published:** 2026-06-30

**Authors:** Rhian Bradley, Afifa Qazi, Sara Casado

**Affiliations:** 1 Kent and Medway Mental Health NHS Trust, Dartford, United Kingdom; 2 Kent and Medway Mental Health NHS Trust, Maidstone, United Kingdom

## Abstract

**Aims:**

The ethos of this project was that regular users of mental health services have complex needs, often falling through the cracks of service provision, which can result in poor and inconsistent care. In the context of our work, these high intensity users (HIU) are defined as having five or more referrals to acute psychiatric services within 90-days. Our aim was to improve the patient experience through working holistically, in a co-produced manner, within a whole system approach, as endorsed within NHS Right Care, Right Person and The Mental Health Crisis Care Concordat.

**Methods:**

Co-production with:

- Staff: HIU leads have been empowered to adapt overarching processes to their local population. Staff feedback has been considered.
- Patients/carers: Focus groups were conducted with lived experience representatives, with ongoing input at system-wide interface meetings.
- External stakeholders: Workshops were conducted with statutory and non-statutory partners.

Resulting in:

- HIU leads in acute and community directorates, supported by clinical lead.
- Multi-agency professional meetings to inform safety planning.
- Specific HIU safety plans, coproduced as therapeutic tools. These plans empower and support clinicians to provide consistent and safe care, with a focus on working with emergency services.
- Monthly, system-wide HIU interface meeting with external partners.
- Information sharing with our system-wide partners.
- Trust wide promotion and resources.

**Results:**

Within the HIU cohort, there was a reduction in the average number of ‘acute’ referrals per month from 80 between August-December 2024, to 28 between August-December 2025. With a notable reduction in 136 referrals from 9 in August 2024, to approximately 2 per month in December 2025. There was also a reduction in the number of admissions to acute wards from an average of 8.8 between August-December 2024, to 3 between August-December 2025, with variation in the average length of stay. There were several ‘good news stories’ of successful collaboration with individuals who were previously struggling to engage with community mental health services. Staff have provided positive feedback, reflecting on the benefits of joint working and that the HIU project is “valuable to get to know patients with long history in services but are not engaging well’.

**Conclusion:**

The partnership has demonstrated value for patients and professional across the system, through providing a coordinated approach to crisis care. Testimonials from partners highlight this: “it shows how Police and mental health services can work together to create a more compassionate, joined-up approach to crisis care, one that listens, responds, and supports rather than reacts or restrains.”

